# *Campylobacter* Colonization, Environmental Enteric Dysfunction, Stunting, and Associated Risk Factors Among Young Children in Rural Ethiopia: A Cross-Sectional Study From the *Campylobacter* Genomics and Environmental Enteric Dysfunction (CAGED) Project

**DOI:** 10.3389/fpubh.2020.615793

**Published:** 2021-01-21

**Authors:** Dehao Chen, Sarah L. McKune, Nitya Singh, Jemal Yousuf Hassen, Wondwossen Gebreyes, Mark J. Manary, Kevin Bardosh, Yang Yang, Nicholas Diaz, Abdulmuen Mohammed, Yitagele Terefe, Kedir Teji Roba, Mengistu Ketema, Negassi Ameha, Nega Assefa, Gireesh Rajashekara, Loïc Deblais, Mostafa Ghanem, Getnet Yimer, Arie H. Havelaar

**Affiliations:** ^1^Department of Environmental and Global Health, College of Public Health and Health Professions, University of Florida, Gainesville, FL, United States; ^2^Emerging Pathogens Institute, University of Florida, Gainesville, FL, United States; ^3^Center for African Studies, University of Florida, Gainesville, FL, United States; ^4^Department of Animal Sciences, Institute of Food and Agricultural Sciences, University of Florida, Gainesville, FL, United States; ^5^Department of Rural Development and Agricultural Extension, Haramaya University, Dire Dawa, Ethiopia; ^6^Department of Veterinary Preventive Medicine, The Ohio State University, Columbus, OH, United States; ^7^Department of Pediatrics, Washington University in St. Louis, St. Louis, MO, United States; ^8^Department of Anthropology, University of Florida, Gainesville, FL, United States; ^9^Department of Biostatistics, College of Public Health and Health Professions & College of Medicine, University of Florida, Gainesville, FL, United States; ^10^Office of Research Affairs, Haramaya University, Dire Dawa, Ethiopia; ^11^College of Veterinary Medicine, Haramaya University, Dire Dawa, Ethiopia; ^12^College of Health and Medical Sciences, Haramaya University, Dire Dawa, Ethiopia; ^13^School of Agricultural Economics and Agribusiness, Haramaya University, Dire Dawa, Ethiopia; ^14^School of Animal and Range Science, Haramaya University, Dire Dawa, Ethiopia; ^15^Global One Health initiative, Office of International Affairs, The Ohio State University, Eastern Africa Regional Office, Addis Ababa, Ethiopia; ^16^Institute for Sustainable Food Systems, University of Florida, Gainesville, FL, United States

**Keywords:** *Campylobacter*, environmental enteric dysfunction, undernutrition, cross-sectional study, Ethiopia, smallholder farming

## Abstract

Livestock farming provides a possible mechanism by which smallholder farmers can meet their household need for animal source foods (ASF), which may reduce the risk of stunting. However, direct/indirect contacts with domestic animals may increase colonization by *Campylobacter* spp., which has been associated with Environmental Enteric Dysfunction (EED) and stunting. A cross-sectional study involving 102 randomly selected children between 12 and 16 months of age was conducted in rural eastern Ethiopia to establish prevalence rates of *Campylobacter* colonization, EED, and stunting, and evaluate potential risk factors. Data were collected between September and December 2018. The prevalence of EED and stunting was 50% (95% CI: 40–60%) and 41% (95% CI: 32–51%), respectively. Among enrolled children, 56% had consumed some ASF in the previous 24 h; 47% had diarrhea and 50% had fever in the past 15 days. 54, 63, 71 or 43% of households owned at least one chicken, cow/bull, goat, or sheep; 54 (53%) households kept chickens indoors overnight and only half of these confined the animals. Sanitation was poor, with high levels of unimproved latrines and open defecation. Most households had access to an improved source of drinking water. The prevalence of *Campylobacter* colonization was 50% (95% CI: 41–60%) by PCR. In addition to the thermotolerant species *Campylobacter jejuni, Campylobacter coli* and *Campylobacter upsaliensis*, non-thermotolerant species related to *Campylobacter hyointestinalis* and *Campylobacter fetus* were frequently detected by Meta-total RNA sequencing (MeTRS). Current breastfeeding and ASF consumption increased the odds of *Campylobacter* detection by PCR, while improved drinking water supply decreased the odds of EED. No risk factors were significantly associated with stunting. Further studies are necessary to better understand reservoirs and transmission pathways of *Campylobacter* spp. and their potential impact on child health.

## Introduction

Undernutrition has been identified as an underlying cause in 45% of under-five mortality globally (1). In Africa, an average of 31.2% of children under-five are stunted, which indicates chronic undernutrition and is represented by length/height for age Z-scores (LAZ/HAZ) of −2 or more standard deviations below median length/height for age of a reference population. Stunted children are short in stature for their age, at increased risk of infectious diseases and vaccine failures, have poor cognitive development, resulting in increased morbidity and mortality and lower lifetime productivity, and income (2–5). In Ethiopia, rates of stunting among children under-five are alarmingly high—up to 38% based on a 2018 report (6). A recent study indicates that average LAZ in a large sample (N = 1,750) of Ethiopian children decreased from −0.7 to −2.0 between 6 and 18 months of age (7). Linear growth faltering during this time period in a child's life therefore not only significantly contributes to the stunting events but also to the overall health status later in life.

Animal-source foods (ASF), including milk, meat, and eggs, have been shown to dramatically decrease undernutrition in children under-five (4, 8). The demand for ASF in Africa is growing rapidly alongside population growth, with projected increases of 145 and 155%, respectively in the demand for meat and milk, between 2005 and 2050 (9). In 2030, egg consumption is projected to be increased by 155% in comparison to 2000 (10). Considering the large increases in the demand of ASF for improving nutritional status and generating income opportunities, the Ethiopian Livestock Master Plan (LMP) aims to significantly increase livestock production in the country (11). However, successful implementation of the LMP will also lead to higher livestock densities in human dwellings and more frequent exposure of human populations to zoonotic pathogens, including *Campylobacter* spp. Next to food and water mediated transmission, direct fecal-oral transmission pathways of zoonotic pathogens to humans are known, mainly through direct contact with animal excreta and contaminated objects in the environment. Occurrence of zoonotic infections may limit the potential benefits of livestock production (7, 12). In Ethiopia, especially the eastern part, chickens, cows, goats, and sheep have close interaction with family members in the homestead. An ethnographic study in the Haramaya woreda (district) in eastern Ethiopia found that chickens and small ruminants are frequently housed in the home overnight with a half-wall barrier separating the livestock animals and the sleeping place of the family members, and frequent contacts between children and farm animals and their contaminated environments occur (13). This cohabitation with livestock creates a distinct opportunity for transmission of zoonotic pathogens from livestock to children (13).

Research highlights that the presence of livestock and their feces may be important risk factors for child malnutrition, particularly stunting (14, 15). The importance of animal feces as a source of exposure to enteric pathogens, causing diarrhea and EED, has been well-documented in recent years. Studies have indicated a strong correlation between poultry ownership and linear growth faltering among children under-five at households. In particular, the overnight presence of chickens within the household was associated with a decrease in HAZ in several countries including Ethiopia (14, 16). A 2017 study found the presence of animal feces to be significantly associated with a lower HAZ score in children 6–24 months of age in Ethiopia (7).

Stunting cannot be completely reversed by optimized diet and reduced diarrhea, leading to the hypothesis that a primary underlying cause of stunting is subclinical gut disease, known as environmental enteric dysfunction (EED) (14). EED is associated with unsanitary disposal of feces, contaminated water supplies, and the presence of domesticated animals in or around the home (14). Increasing evidence suggests that repeated or persistent asymptomatic (i.e., without diarrheal illness) colonization with enteric pathogens may result in EED and stunting (15, 17). Therefore, assessment of the colonization of children with enteric pathogens, or more broadly full characterization of the gut microbiota, is critical to further understanding the complex interactions between pathogens, the environment, and human hosts ultimately leading to malnutrition and other poor health outcomes in children.

Poultry and other warm-blooded animals are the natural reservoirs of thermophilic *Campylobacter* spp. (18). Humans and other higher primates may develop disease after intestinal colonization with *Campylobacter* spp. (19). Diarrheal disease (the most commonly observed), and autoimmune diseases, as well as digestive disorders, may be triggered after infection with *Campylobacter* spp. (20). A study in Peru indicated that asymptomatic and symptomatic *Campylobacter* infections were associated with reduced weight gain over a three-month period, and symptomatic *Campylobacter* infections were marginally associated with reduced linear growth over a nine-month period (21). A recent multi-center study identified a high prevalence of *Campylobacter* spp. in stool samples of primarily asymptomatic children in low-resource settings being associated with a lower LAZ score and increased level for markers of EED (intestinal permeability and intestinal and systemic inflammation) at 24 months of age (15). Thermotolerant species including *Campylobacter jejuni* and *Campylobacter coli* are the most frequent causes of bacterial enteritis globally (22). Non-thermotolerant *Campylobacter* species (e.g., *Campylobacter hyointestinalis, Campylobacter fetus*, and *Candidatus Campylobacter infans*) are a diverse group of bacteria, whose pathogenic mechanism is not-well-established (23). In a follow-up study, fecal samples were tested with quantitative polymerase chain reaction (PCR) for a large array of enteropathogenic organisms, and *C. jejuni/C. coli* were among a limited number of pathogens significantly associated with decreased LAZ, and the only pathogens maintaining this consistent trend in a longitudinal model (17). A causal role of *Campylobacter* spp. in the pathology of EED has been recently observed in a mouse model. When diets were deficient in zinc, consistent bloody diarrhea and weight loss were observed, as well as elevation of fecal inflammation markers of EED including fecal myeloperoxidase (MPO) in mice infected by *C. jejuni* (24).

These lines of evidence converge to suggest that *Campylobacter* species, a natural inhabitant of the gastrointestinal tract of livestock and poultry, are among the main pathogenic bacteria involved in the causal chain of stunting. Nevertheless, in Ethiopia, little research has been conducted to estimate the reservoirs or transmission pathways of *Campylobacter* colonization. The association of *Campylobacter* with EED and stunting, due to young children's direct or indirect exposure to feces of these animals, and socio-demographic factors that may affect this association are not well-understood. To fill these knowledge gaps, the formative research of the project, *Campylobacter* Genomics and Environmental Enteric Dysfunction (CAGED), includes a cross-sectional epidemiological investigation involving young children to estimate the prevalence and species diversity of *Campylobacter* spp. in gut microbiota in relation to the prevalence of EED and stunting. Results of PCR and metagenomic analyses of gut microbiota, reflecting *Campylobacter* species prevalence along with microbial diversity among the young children are presented in a companion manuscript (25). Here we aim to better understand the relationships of *Campylobacter* colonization in the intestine of children with the occurrence of EED and stunting and divulge potential risk factors in their socio-demographic context.

## Materials and Methods

### Study Design and Population

A community-based cross-sectional study, including a questionnaire-based surveillance of the households and collection of child growth data, child urine, and fecal samples were performed in the Haramaya woreda (district), East Hararghe Zone, Oromia Region, Ethiopia. Beginning in 2018, Haramaya University (HU) initiated a full household enumeration of 12 kebeles (wards) within Haramaya woreda using methods developed for the Kersa Health and Demographic Surveillance System (26). For this cross-sectional study, five out of the 12 kebeles were selected in a way to capture the spatial heterogeneities in the landscape (e.g., land use, land cover, altitude, agricultural practices) that could influence risk factors contributing to health outcomes.

Eligibility criteria of this study were designed to enroll children receiving traditional maternal care and without underlying health conditions that might affect the primary study outcomes. Previous studies (MAL-ED) have indicated that the prevalence of *Campylobacter* spp. in children in low- and middle-income countries (LMIC) increases linearly in the first year of life and then reaches a steady state or slow decline with peak levels occurring around 12 months of age (15). A child was included in the study if he/she was 11–13 months of age when consent was given, and if the child's mother was the caretaker. The child was excluded from the study if he/she presented visible congenital abnormality or had serious medical illnesses, or the child or his/her mother required extended stay in hospital after birth. Households with at least three chickens within the homestead (defined as the small collection of households that are physically connected to one another), willing to participate and conform to the requirements of the study were included. Families not residing in Haramaya woreda for at least 3 months, currently participating in another study on animal husbandry, or with a mother who did not live in Haramaya woreda when the child was born were excluded.

The sample size of this study considered estimation of the three primary outcomes: *Campylobacter* colonization, EED, and stunting. Based on a binomial distribution, a sample size of 100 for the whole study population allowed estimation of a 50% prevalence with precision of 10 at 95% confidence (R function *binom:: binom.confint*).

While adjusting for population weight of kebeles, 102 children (one child from each household) were randomly selected from the sampling frame of the selected five kebeles. Field work started on October 16, 2018 and continued for 10 weeks until December 21, 2018. The enrolled children and their mothers were invited to come to a local health post or administration office for anthropometric measurements, administration of a sugar solution, and collection of a urine sample for each child for later measurement of lactulose excretion (a marker for EED in urine), as well as completion of interviews and providing a fecal sample for each child.

### Field Activities

Questionnaires were developed in collaboration with the Haramaya University (HU) field team using validated indices whenever possible, such as minimum dietary diversity of infant and young children (MDD-IYC). In consultation with social scientists and field workers, the study questionnaire was finalized so that it was culturally appropriate and locally adapted. In the local language (Afan Oromo), bilingual data collectors collected information on demographics, livelihoods, wealth, animal ownership, animal management and disease, water, sanitation and hygiene, health, and nutrition. Study data were collected and managed using REDCap electronic data capture tools hosted at the University of Florida Clinical and Translational Science Institute (27).

### Collection and Analyses of Stool and Urine Samples

During the implementation of household surveillance, stool samples from the selected children were also collected aseptically in a sterile plastic sheet for EED and PCR analyses, and meta-total RNA sequencing (MeTRS, a culture-independent sequencing method that profiles the presence and quantity of RNA transcripts in a sample). Samples were flash-frozen in the field using liquid nitrogen and stored at −80°C until further use. Genomic DNA and RNA were extracted for conventional PCR and MeTRS, respectively (25). Details of laboratory procedures on PCR and MeTRS were described in Terefe et al. (25). For the dual sugar absorption test, an oral solution consisting of 200 mg L-rhamnose and 1000 mg lactulose in 10 mL sterile water was prepared as previously described (28). Study personnel administered the entire solution to each child over a period of 5 min. A urine bag was then placed on the child 30 min after consuming the solution and all urine collected over the subsequent 60 min was returned to the data collector. Analysis of lactulose and rhamnose was conducted according to a previously described procedure (29, 30). In stool samples, myeloperoxidase (MPO) was measured using a commercially available enzyme-linked immunosorbent assay (MPO RUO, Alpco, Salem, NH). One child had severe diarrhea and difficulty in producing feces, we were unable to collect a fecal sample from him; another child produced a small amount of feces that was only sufficient for PCR testing. The samples sizes for PCR, MeTRS, and MPO analyses were therefore 101, 100, and 100, respectively.

Threshold cutoffs for moderate and severe EED based on the dual sugar absorption test and fecal MPO were defined following previously described methods (31, 32). As reported by Ordiz et al. (31), urinary excretion of lactulose and rhamnose were highly correlated as illustrated in Supplementary Figure 1, and following these authors, we adopted the percentage of lactulose excretion as a biomarker for gut permeability associated with EED. Cutoff values for the percentage of lactulose (%L) were as suggested by Agapova et al. (33): 0.2 < %L ≤ 0.45% for moderate EED, and %L > 0.45 for severe EED. Following Kosek et al. (32), we defined a threshold for fecal MPO of 2,000 ng/ml for gut inflammation and the third quartile of the observed data (11,000 ng/ml in our cohort) as a threshold for severe inflammation.

### Anthropometric Measurements

The child's weight, recumbent length, and Mid-Upper Arm Circumference (MUAC) measurements were collected by trained caregivers using Seca 334 baby scales, Seca 417 length boards (Itin Scale Co., Inc., Brooklyn, NY), and MUAC tape precise to 0.01 kg, 0.1, and 0.2 cm, respectively. Length measurements were taken three times. The average length was calculated by taking the arithmetic mean of the data after outlier removal as described in Supplementary Appendix 1. Length-for-age, weight-for-age, and weight-for-length Z scores (LAZ, WAZ, and WLZ, respectively) were calculated according to WHO Child Growth Standards using R package *anthro*. Severe acute malnutrition was defined according to Ethiopian clinical standards (MUAC <110 mm and length >65 cm or a WLZ ≤ −3) (34).

A total of 12 children with one (or more) of the following medical condition(s) were referred to a healthcare facility, including four cases of dehydration, five cases of diarrhea and fever, and six cases of diarrhea. Two children were referred to a health post for additional follow-up for severe acute malnutrition.

### Data Analysis

Primary outcome variables of interest were *Campylobacter* colonization (by PCR), EED, and stunting. Descriptive analysis was undertaken to explore background features of the study population. 95% binomial proportion confidence intervals for prevalence were based on Wilson score. Explanatory variables potentially associated with the outcome variables were selected based on *a priori* knowledge (1–4, 7, 8, 10, 12, 13, 16). All data analyses were performed in the statistical language R version 3.5.1 (35).

Composite variables were generated to assist in analyzing the sociodemographic data. We created Water, Sanitation, and Hygiene (WASH) ladder variables (i.e., drinking water, sanitation, and hygiene ladders) based on the Joint Monitoring Program (JMP) by WHO and UNICEF (22, 36). Tropical livestock unit (TLU), a composite metric for quantifying all farming animals in a household, was calculated following published literature (37).

Bivariate and multivariate analyses were conducted to explore associations between the three outcome variables and potential explanatory variables using unadjusted and adjusted logistic regression models [controlling for child's age (in days), sex, and kebele of residence]. To strengthen the robustness of the models, continuous [i.e., child's and mother's age, TLU, household wealth (asset and income)] and ordinal explanatory variables were, respectively dichotomized by medians or generally accepted cut-offs (i.e., minimum dietary diversity score of 5). We established multiple exposure models by including the three confounders (child's age group, sex, and kebele of residence) and candidate variables with *p*-values < 0.20 from the adjusted single exposure models (Supplementary Tables 4–6), and backward stepwise selections were conducted to sort out the significant explanatory variables (38). Given the small sample size, all *p*-values and 95% confidence intervals (CIs) were estimated by likelihood-ratio (LR) test, and we did not include interaction between the explanatory variables in the models. Observations with missing values were excluded from the analyses.

## Results

### Sociodemographic Characteristics

#### Child Health and Diet

At the time of data collection, the mean age of children was 428 days (SD = 31, range 360–498 days). As reported by mothers, nearly half (47%) of children had experienced diarrhea (defined as three or more loose or watery stools) in the previous 15 days, while 12% currently had diarrhea in the previous 24 h; half (50%) of children experienced fever in the previous 15 days, with 5% currently having fever in the previous 24 h (Table 1). Based on the caregiver survey, 35% of the children encountered both fever and diarrhea in the previous 15 days, and 3% currently had both symptoms.

**Table 1:** Child health and diet in five rural kebeles in Haramaya woreda, Eastern Ethiopia.

	**Number of children (%)**
Sex of child, male (*n* = 102)	50 (49%)
Child only had diarrhea in past 15 days (*n* = 102)	48 (47%)
Current diarrhea^b^	12 (12%)
No current diarrhea^b^	36 (35%)
Child only had fever in past 15 days (*n* = 101^a^)	51 (50%)
Current fever^b^	5 (5%)
No current fever^b^	46 (45%)
Child had both fever and diarrhea in past 15 days (*n* = 101^a^)	35 (35%)
Current diarrhea and fever simultaneously^b^ (*n* = 101^a^)	3 (3%)
Child consumed any animal source food (ASF) in last 24 h (*n* = 102)	57 (56%)
Child consumed eggs in the past 24 h (*n* = 102)	5 (5%)
Child currently breastfed (n=100^a^)	90 (89%)
Child exclusively breastfed through 6 months (*n* = 96^a^)	50 (52%)
Breastfed immediately at birth (*n* = 101^a^)	100 (99%)

a *The variation of n on this table is due to missing values*.

b *The symptom was experienced in past 24 h*.

Within the sample population, 99% of children were breastfed at birth and 89% were still breastfed at the time of data collection (Table 1). The rate of exclusive breastfeeding through 6 months was low, at 52%, indicating that just under half of the children had received supplementary food other than breastmilk to eat or drink prior to 6 months of age (36) (Table 1).

According to 24-h food recall by mothers, only 9% of children met the minimum dietary diversity threshold for infants and young children (MDD – IYC) of consuming five out of eight food groups (note: breastmilk is included in this measure). However, 56% (57/102) of children had consumed animal source food, of whom a strong majority (93%, 53/57) consumed milk and relatively few (9%, 5/57) consumed eggs. Consumption of goat's milk by children is not very common in the area and heat treatment of milk is uncommon in rural areas. Thus, reports of milk being consumed by young children are most likely unpasteurized cow's milk, though this was not specified in the questionnaire. No child was reported to have consumed any animal meat or flesh product. Most children consumed 3–4 food groups (range 1–6 food groups), mainly from the food groups of (1) grains, roots, and tubers, (2) legumes and nuts, and (3) breastmilk.

#### Water, Sanitation, and Hygiene (WASH)

Sanitation, drinking water, and hygiene ladders indicate very low levels of WASH conditions. Only 23% of households used improved sanitation facilities shared between households (limited), and 77% of households practiced open defecation (Sanitation Ladder, Table 2). We observed 56% of respondents reported drinking water from an improved source that required <30 min for a roundtrip collection. 42% of respondents also had access to an improved source, however it required more than 30 min to collect, while only 2% reported having access to a safely managed drinking water source located on premises, available when needed, and not contaminated by fecal and chemical pollutants (see Drinking Water Ladder, Table 2). Among women, 93% reported having no place to wash their hands, while 7% had a facility, but without soap and water.

**Table 2:** Water, sanitation and hygiene indicators in five rural kebeles in Haramaya woreda, Eastern Ethiopia.

	**Number of children (%)**
Sanitation ladder (*n* = 102)	
Open defecation	79 (77)
Unimproved	0
Limited	23 (23)
Basic	0
Safely managed	0
Drinking water ladder (*n* = 99^a^)	
Surface water	0
Unimproved	0
Limited	42 (42)
Basic	55 (56)
Safely managed	2 (2)
Hygiene Ladder (female) (*n* = 101^a^)	
No handwashing facility	94 (93)
Limited facility on premises without soap and water	7 (7)
Basic facility on premises with soap and water	0

a *The variation of n on this table is due to missing values*.

#### Animal Husbandry

Most families (94%) owned some livestock, typically the equivalent of 2-3 TLU, and 54, 63, 71, and 43% of households had at least one chicken (range 0–15 chicken), one cow/bull (range 0–7 cattle), one goat (range 0–10 goat), and one sheep (range 0–7 sheep), respectively. Very few other animals were reported, including cats, dogs, camels, and others.

Of the 54% of households who reported owning chickens, all but one respondent reported keeping their chicken(s) within the house at night. To characterize the increasing likelihood of contact between chickens and study participants based on poultry management practices, we defined a Chicken Nighttime Location risk score as elucidated in Table 3. Households who either had no chickens or did not keep them in the home at night (46%) were assigned a chicken nighttime risk score of 0; another 26%, who kept their chickens in the home at night but reported *confining* the chicken within the household, were assigned a risk score of 1; and the remaining 28%, who kept their chicken in the home *without confinement*, were assigned a risk score of 2 (Table 3).

**Table 3:** Chicken husbandry for sampled households in five rural kebeles in Haramaya woreda, Eastern Ethiopia.

	**Number of households (%)**
Chicken hoarding status at night (*n* = 101)	
Household does not have chickens	46 (46)
Kept outside homestead during night	1 (1)
Kept inside homestead, but outside house during night	0
Kept inside house during night	54 (53)
Confined inside house at night	26 (26)
Not confined inside house at night	28 (28)
Chicken nighttime location risk score (*n* = 101)	
0 (no chickens, or not kept inside at night)	47 (46)
1 (chickens kept in house at night, confined)	26 (26)
2 (chickens kept in house at night, unconfined)	28 (28)

### Campylobacter Colonization

*Campylobacter* prevalence in child fecal samples was 50% (95% CI: 41–60%, *n* = 101) as deduced from PCR-based detection (23), and 12% of the children were reported to have current diarrhea. MeTRS revealed 88% (95% CI: 80–93%, *n* = 100) prevalence of the genus *Campylobacter* among the studied samples. Eight *Campylobacter* species showed the highest prevalence: *C. jejuni, C. hyointestinalis, C. coli, Campylobacter* sp. RM6137, *C. upsaliensis*, an uncultured *Campylobacter* sp. and *Campylobacter* sp. RM12175 (Table 4). Interestingly, most of the children (82%, *n* = 100) were observed to be infected with multiple species of *Campylobacter*.

**Table 4:** *Campylobacter* species detected in child fecal samples by Meta-total RNA sequencing (MeTRS) in five rural kebeles in Haramaya woreda, Eastern Ethiopia.

**Species**	**Prevalence^b^ (*n* = 100)**	**Mean log_10_(RPM^a^)**	**SD log_10_(RPM)**
*Campylobacter* genus	0.88	3.42	0.98
*Campylobacter jejuni*	0.68	2.19	1.12
*Campylobacter hyointestinalis*	0.65	2.75	1.13
*Campylobacter coli*	0.62	1.76	1.16
*Campylobacter* sp. RM6137^c^	0.53	2.08	1.00
*Campylobacter upsaliensis*	0.50	1.79	1.03
*Uncultured Campylobacter* spec.	0.46	1.85	1.05
*Campylobacter* sp. RM12175^c^	0.41	2.79	1.24

a *Abundance (Reads Per Million)*.

b *The table is ordered by prevalence. Only species with prevalence >0.40 and mean log_10_(RPM) >1.5 are included*.

c *Belongs to the Campylobacter fetus group*.

### EED

As expected, absorption of lactulose ($\bar{\%L}$ = 0.27) and rhamnose ($\bar{\%R}$ = 4.03) are highly correlated (Pearson's ρ= 0.44, *P*-value < 0.01) (Supplementary Table 1 and Supplementary Figure 1). Based on the specified threshold cutoffs for MPO and %L biomarkers, the prevalence of EED in the study children was deduced to be 50% (95% CI: 40–60%, *n* = 100), among whom 17% (95% CI: 11–26%) had severe EED (Supplementary Table 2).

### Anthropometry

**Figure 1:**
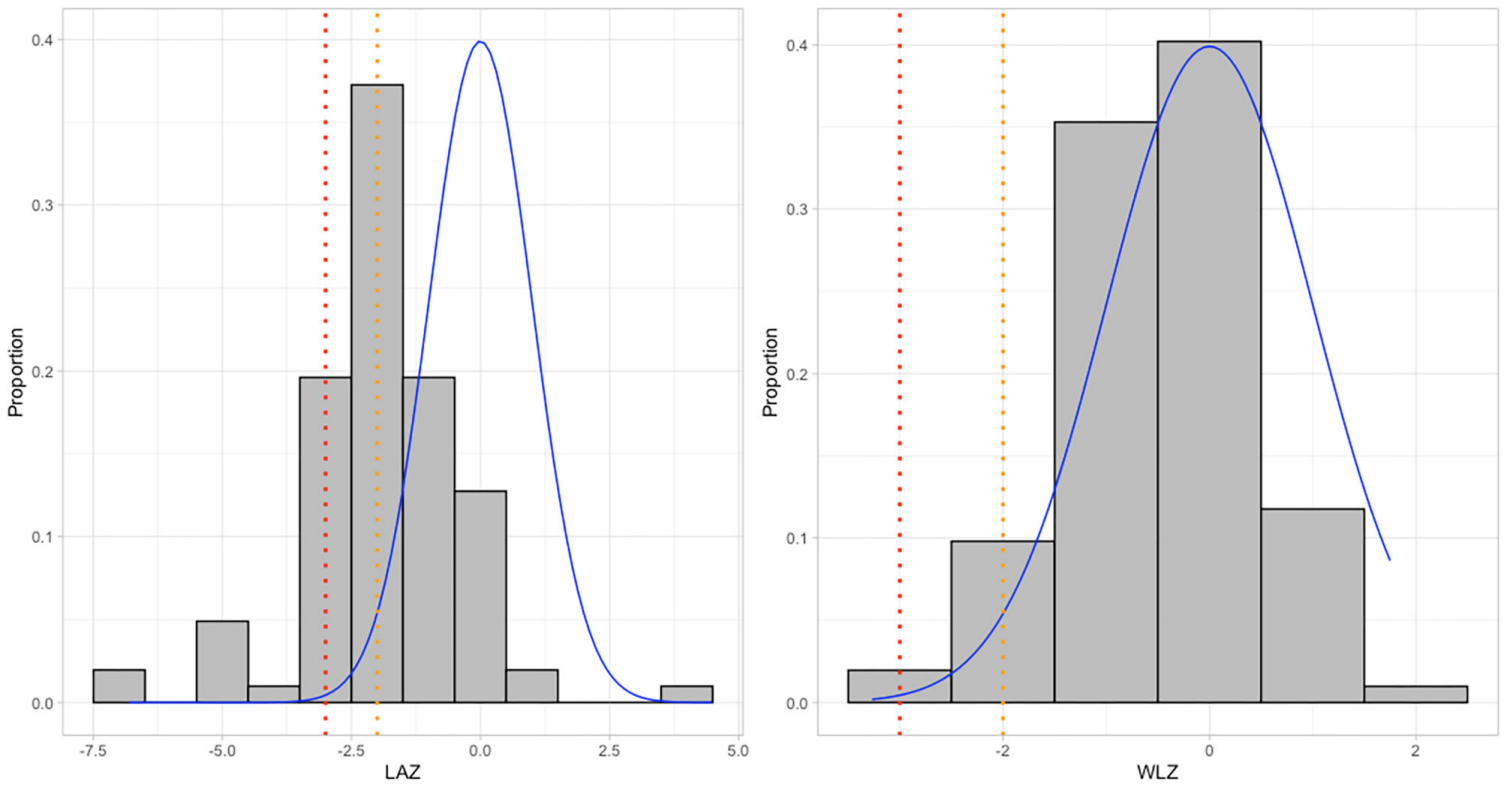
Distribution of LAZ & WLZ compared to WHO normal growth standards in five rural kebeles in Haramaya woreda, Eastern Ethiopia. The histograms show the distribution of LAZ and WLZ in the sample; the blue curve shows a standard normal distribution of WHO growth reference (μ = 0, σ = 1). Orange and red dotted lines indicate thresholds for stunting/wasting (Z score <-2) and severe stunting/wasting (Z score <-3), respectively.

Comparing with the reference distribution of LAZ and WLZ (both with a mean = 0 and SD = 1), the full distribution of LAZ values was shifted to the left, while the distribution of WLZ had a mode at 0 but was tailed to the left (Figure 1). The means for LAZ, WAZ, WLZ, and MUAC were −1.88 (SD = 1.56), −1.25 (SD = 1.18), −0.42 (SD = 0.91), and 130 mm (SD = 11), respectively (Supplementary Table 3). Stunting was observed in 41% (95% CI: 32–51%) of the study population, while severe stunting occurred in 19% (95% CI: 12–27%) of the children (Table 5). Wasting and severe wasting were observed in 5% (95% CI: 2–11%) and 1% (95% CI: 0–5%) of the population, respectively, and 3% (1–8%) of children were both stunted and wasted (Table 5).

**Table 5:** Stunting and wasting levels in five rural kebeles in Haramaya woreda, Eastern Ethiopia.

	**Number of children**	**Prevalence (95% CI)**
Stunting (*n* = 102)		
Not stunted (LAZ ≥−2)	60	59% (49–68%)
Stunted (LAZ < −2)	42	41% (32–51%)
Severely stunted (LAZ < −3)	19	19% (12–27%)
Wasting (*n* = 102)		
Not wasted (WLZ ≥−2)	97	95% (89–98%)
Wasted (WLZ < −2)	5	5% (2–11%)
Severely wasted (WLZ < −3)	1	1% (0–5%)
Stunted and wasted (*n* = 102)		
(LAZ < −2 & WLZ < −2)	3	3% (1–8%)
Underweight (*n* = 102)		
Not underweight (WAZ ≥−2)	75	74% (64–81%)
Underweight (WAZ < −2)	27	26% (19–36%)
Severely underweight (WAZ < −3)	8	8% (4–15%)
Acute malnutrition (*n* = 102)		
No (MUAC ≥ 125 mm)	71	70% (60–78%)
Yes (MUAC < 125 mm)	31	30% (22–40%)
Severe (MUAC < 115 mm)	8	8% (4–15%)

### Risk Factor Analyses

**Figure 2:**
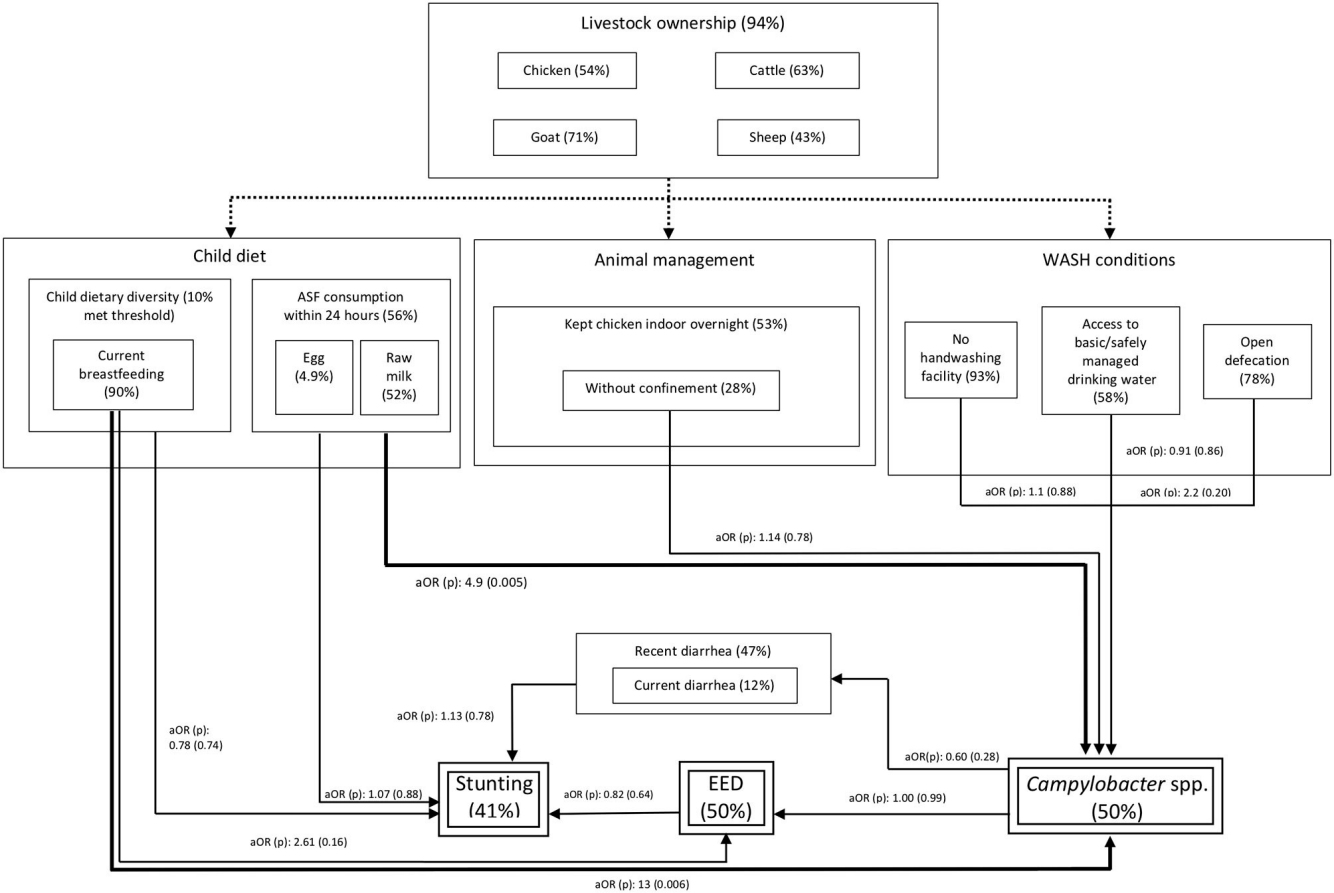
Putative relationships between animal ownership and management, WASH conditions, diets and the main outcome variables in this study: colonization of children with *Campylobacter* spp., environmental enteric dysfunction (EED) and stunting. The rounded-dot dash lines represent the complex interactions between livestock ownership and risk factors explored in this study. The thick bold lines represented significant (*p* < 0.05) associations in the multivariate models. The thinner lines represent tested associations in the single exposure models. aOR: adjusted odds ratio (*p*-value in parenthesis).

Supplementary Tables 4–6 present crude and adjusted odd ratios, and Table 6 depicts the models with single (with p [probability]-values <0.2) and multiple risk factor(s), i.e., the *Campylobacter* model considering children's current breastfeeding and animal source food consumption practices as risk factors. The five kebeles could be categorized into three sets by assembling the three kebeles with similar prevalence of *Campylobacter* (by PCR) into a new group as a confounder for kebele effect in *Campylobacter* models (Supplementary Table 4). Given this three-level kebele effect did not yield statistically different prevalence of EED and stunting (Supplementary Tables 5, 6), this scheme was kept in the models of these two outcomes (i.e., EED and stunting) to account for spatial heterogeneity. Statistical analysis showed that the group of children who are being breastfed and also currently consuming ASF had significantly higher odds for the presence of *Campylobacter* spp. when adjusting for confounders and other risk factors at the 5% level (Table 6). EED was treated as a binary variable by combining categories of moderate and severe EED as listed in the Supplementary Table 2. The household's access to a basic or safely managed drinking water source significantly reduced the odds of EED at the 5% level (Table 6). No significant crude or adjusted associations were found between stunting and any of the explanatory variables (Supplementary Table 6), hence multiple exposure models were not constructed. Also, the aforementioned associations (measured through the logistic regressions) between the explanatory factors and the three health endpoints (i.e., colonization with *Campylobacter* spp., EED, and stunting) and their prevalence estimates are depicted in Figure 2.

**Table 6:** Univariate and multivariate models for *Campylobacter* and EED outcomes of children in Haramaya woreda, Eastern Ethiopia.

	**Unadjusted**		**Adjusted**^**b**^	
	**OR (95%CI^**a**^)**	***p*-value^**a**^**	**OR (95%CI^**a**^)**	***p*-value^**a**^**
***Campylobacter*** **spp. occurrence (by PCR)**				
**Model with single risk factor**				
Currently breastfed^c^	2.98 (0.80,14.30)	0.10	9.50 (1.47,85.47)	0.02
Animal source food consumption in last 24 h^c^.	1.43 (0.65,3.18)	0.37	4.01 (1.35,14.00)	0.01
Egg consumption in last 24 h^c^.	4.17 (0.59,83.19)	0.16	5.87 (0.73,124.51)	0.10
**Model with below multiple risk factors**				
Currently breastfed^c^	-	-	13.36 (2.01,138.61)	0.006
Animal source food consumption in last 24 h^c^.	-	-	4.87 (1.58,17.76)	0.005
**EED**				
**Model with single risk factor**				
*Campylobacter* by MeTRS[log_10_(RPM)]^d^	2.17 (0.98,4.92)	0.06	2.29 (0.98,5.50)	0.06
Currently breastfed^c^	1.83 (0.52,7.41)	0.35	2.61 (0.69,11.35)	0.16
Exclusive breastfeeding through 6 months^c^	1.84 (0.81,4.23)	0.14	2.46 (1.00,6.40)	0.05
Mother's age^d^	1.84 (0.83,4.13)	0.13	2.2 (0.96,5.26)	0.06
Animal source food consumption in last 24 h^c^.	0.45 (0.49,2.40)	0.06	0.51 (0.61,3.58)	0.12
Basic or safely managed drinking water^e^	0.33 (0.14,0.76)	0.009	0.34 (0.14,0.81)	0.02
Chickens in home at night^c^	1.84 (0.83,4.15)	0.13	1.95 (0.86,4.57)	0.11

a *Estimated by likelihood-ratio (LR) test*.

b *Adjusted for child age (in days), sex and kebele group*.

c *Reference group: population without having the risk factor*.

d *Reference group: population with the continuous values less than median*.

e *The ORs were calculated by taking the limited access group as reference*.

## Discussion

The socio-demographic context in rural Ethiopia and the complex, multifactorial eco-pathological processes that influence the colonization by *Campylobacter*, occurrence of EED, and impaired growth among children make it challenging to identify causative factors and exposure routes of the pathogen. Nevertheless, the findings from the household surveys are useful in understanding the high prevalence of malnutrition, poor gut health, and *Campylobacter* colonization of young children. Within the complex network of pathogens exposures, animal husbandry and the presence of domestic animals have been shown to be associated with higher burdens of *Campylobacter* colonization, acute disease (e.g., diarrhea, fever), and chronic outcomes such as EED and stunting. The results of this study also indicate that the detrimental effects to child health of insufficient WASH and poor animal husbandry conditions are likely exacerbated by poor diets. The strengths of our study include the application of mixed-method and following of the “One Health” approaches, allowing transdisciplinary and multisectoral teams to collaborate.

### Undernutrition

According to the 2018 Global Hunger Index, Ethiopia ranks 93rd out of 199 qualifying low/middle income countries and suffers from a level of hunger that is considered serious (39). The prevalence of stunting in children under 5 years of age has decreased from 57.4% in 2000 to 38.4% in 2018. In the study area, we found that the prevalence of stunting among children 12–16 month of age is 41% (95% CI: 32–51%), which is higher than a 2020 study in southern rural Ethiopia (31.4%) (40). Conversely, our study found a lower rate of wasting 5% (2–11%) compared to the southern rural region in this country (14.3%) (40). On the other hand, in rural Ethiopia, average HAZ value has been reported to be −1.7 at approximately 9.6–20 months of age (7), whereas the average LAZ in our setting at that age was −1.9. Hence, chronic malnutrition in the area of this study appears to be more serious than the country's rural average.

### *Campylobacter* Species Diversity and Risk Factors

The prevalence of *Campylobacter* spp. among almost 50% of the under-five children in our setting is similar to Tanzania from the MAL-ED study and another recent surveillance study conducted in southern rural Ethiopia (15, 40), when measured by PCR, confirming a high burden of *Campylobacter* colonization. High exposure to pathogenic microorganisms is also indicated by high prevalence of diarrhea and fever (41, 42). The results of this study aligns with findings from previous studies that indicate a considerable number of diarrheal episodes can be associated with *Campylobacter* spp., which is one of the most commonly detected species in stool samples in Peru, South Africa, Brazil, and Nepal (41, 42). The metagenomic results confirmed that the thermotolerant *C. jejuni* and *C. coli*, which are commonly associated with chickens and livestock, are frequently colonizing at high abundance levels in the gut of young children, that we have also presented in more details (18). Notably, we observed the presence of *C. upsaliensis*, which is a member of the *C. jejuni* group but commonly associated with cats and dogs. An important finding of our study is the occurrence of a large number of other, non-thermotolerant *Campylobacter* species known to be highly diverse. Their reservoirs are poorly characterized, especially in low- and middle-income countries. The frequent presence of these non-thermotolerant species, including *C. hyointestinalis*, showing higher abundance than *C. jejuni/C. coli*, indicates a salient impact of various animals on child health in Ethiopia. Previous studies have shown that intestinal colonization of C. *upsaliensis, C. fetus*, and *C. hyointestinalis* can induce gastroenteritis in humans (43–45). Apart from human isolates, *C. hyointestinalis* subsp. *hyointestinalis* has been mainly isolated from ruminants while *C. hyointestinalis* subsp. *lawsonii* has been isolated from pigs (46). The primary reservoirs of *C. fetus* subsp. *fetus* include cattle and sheep, but this subspecies has also been isolated from the feces of other animal species. Among the *Campylobacter* spp. identified by MeTRS in this study, *C. fetus* subsp. *venerealis* is a cause of infection in cows (44), and *Campylobacter* sp. RM 6137 and *Campylobacter* sp. RM12175 are unnamed novel species in the *C. fetus* group that have been isolated from feces of wild pigs and cows, respectively (47). Therefore, frequent occurrence of *C. jejuni*/*C. coli* in children supports the hypothesis that chickens are a main reservoir of these bacteria. The presence of other species of *Campylobacter* implies that domestic and farmed animals other than poultry may also significantly contribute to environmental exposure and colonization of children. This inference is further supported by the increased risk of *Campylobacter* colonization associated with ASF consumption, which in this region mainly includes raw cow's milk (Table 6).

The observed significantly increased risk of *Campylobacter* colonization associated with current breastfeeding, which is in accordance with a growing body of evidence indicating that exclusive breastfeeding may provide a gut environment, more favorable to growth of (certain species) of *Campylobacter* (23, 48). It is currently not clear how breastfeeding provides a competitive advantage to *Campylobacter* species in the infant gut (23). Nonetheless, the significance and importance of breastfeeding for overall child infant nutrition, development, and protection against infectious diseases is well-documented (48). Our findings should not be interpreted as to discourage breastfeeding of infants and young children but underscore the importance of controlling *Campylobacter* exposure, as well as overall gut health, to protect and ensure proper growth of children.

### EED

There is no gold standard for classifying EED based on biomarkers, and different approaches have been used to define this endpoint (28, 32). We adopted the Lactulose: Rhamnose test, which is now considered more appropriate than the Lactulose: Mannitol test as a marker for intestinal permeability, and MPO as a well-established marker of neutrophil activity and gut inflammation (28). A classification based on these two markers was then used to define moderate and severe EED and revealed a high burden of EED in our study area. Fecal MPO results are known to be strongly variant within and between populations geographically. The median MPO concentration in our setting is lower than the median of all sites in the MAL-ED cohort (32). This does not necessarily imply a lower EED burden in our study population, because the variance of MPO may be due to different age of children in the two studies, since MPO is age-dependent (49).

### Child Health and Nutrition

Typically, the diarrheal prevalence has been reported to be 20–25% among the under-five children in the study region (50, 51). However, the mother interviewed in our study have reported a higher prevalence (48%) of recent diarrhea (in the previous 15 days) among the children aged between 12 and 16 months. A high prevalence of recent diarrhea in children, as reported by their mothers, has also reported from Pakistan (63%), Bangladesh (53%), and Peru (53%), whereas lower prevalence was reported in India (21%), Nepal (27%), South Africa (4%), Tanzania (6%), and Brazil (5%) (15). These differences in diarrheal prevalence might be due to variations in selection criteria, geo-climatic conditions, and WASH and feeding practices. For example, in congruence to our study, increased diarrhea has been found in populations consuming more milk in southeast Asia (52). Further investigation of the microbial content of ASF, as well as more careful characterization of symptomatic diarrhea would aid in better understanding this potential driver of stunting among different child cohorts.

Only 9% of children in our study population met the MDD-IYC of five out of eight food groups (53). While intake of diverse ASF can significantly improve child diet quality, no child consumed any animal flesh, only about half of children consumed dairy products, and very few of them consumed eggs. Though seasonality of diet may vary significantly in parts of Ethiopia (54), evidence does not suggest that these data reflect lower than normal rates of MDD-IYC than would otherwise be observed in the study area. Moreover, increased diversity in ASF for younger children may vary seasonally with parents income (55), which was not captured in this study.

### Influence of WASH Status and Livestock Management

Our fieldwork indicated that people in the Haramaya woreda do not consider exposure to animal feces a public health issue, and household members in the community had little knowledge or concern about the risks of zoonotic exposure. In the study region, access to or use of proper sanitation facilities was observed to be very poor and human and/or animal excreta were found scattered in most homesteads. Despite the general suboptimal drinking water supply, we found access to the basic or safely managed source of drinking water was associated with reduced odds of EED. This emphasizes the necessity of improving the drinking water supply in the study region. Moreover, people's perception of the significance and adequate practice of hand-washing was low in general. The above contexts of WASH practices could not only induce microbial exposure (e.g., *Campylobacter* spp.) to the mothers but also to their children.

Most of the study families had their own livestock and poultry, and most animals were freely roaming around without proper management of animal droppings. As observed, most of the families kept the smaller animals in house overnight due to security concerns; in over half of households, chickens roamed freely within household compounds during nighttime. This kind of animal husbandry, in addition to the poor status of WASH practices, place human populations at high risk of exposure to zoonotic pathogens and may explain the observed high prevalence of *Campylobacter* and EED in children. Nonetheless, other research underlines the difficulty of unraveling these complex networks: despite multiple observational studies that found household-level WASH to be strongly associated with child linear growth, three landmark randomized clinical trials found WASH interventions had no statistically significant impact on the linear growth, even though effective behavior change and prevalence reduction for some enteric pathogens were achieved (56, 57).

### Limitations

Only a few significant associations between explanatory variables and stunting, EED, and *Campylobacter* colonization among young children could be ascertained, which might be due to relatively small sample size of this study. Other limitations of this study included the use of the cross-sectional survey data to evaluate variation in health status as a consequence of multiple factors interacting on different time scales, including children's diet and breastfeeding practices, and their health conditions (diarrhea and fever statuses), which are complex interactions particularly susceptible to recall bias (58).

## Conclusion

Our study reveals high burdens of *Campylobacter* colonization, EED, and stunting in the traditional rural Ethiopian settings of smallholder farmers. Results of this study indicate that child health in this rural setting may be negatively influenced by poor diversity of diet despite high level of breastfeeding, low level of WASH, in combination with poor livestock management. Common occurrence of emerging *Campylobacter* spp. was also observed. High prevalence of stunting and undernutrition among young children in this region may be due to chronic exposure to *Campylobacter* infection. A variety of *Campylobacter* spp., including thermotolerant *C. jejuni, C. coli*, and *C. fetus* and non-thermotolerant *C. hyointestinalis* species, which commonly occur in not only poultry and livestock but also in pets and wild animals, were observed to occur in high prevalence among young children, many of whom suffering from frequent diarrhea. Our study contributes to a body of literature underscoring the necessity of considering “transformative WASH” in future research to decipher the multifaceted causal pathways between zoonotic pathogens and child growth faltering and better understand how the colonization of children with *Campylobacter* spp. is related to EED and stunting, against a background of poor WASH and nutritional conditions. Further, a longitudinal study is planned to better understand animal reservoirs and transmission pathways for colonization of children with *Campylobacter* spp., inducing EED and stunting, against a background of poor WASH and nutritional conditions. In the same study population, an exposure assessment investigation following the Sanipath methodology will be embedded within the longitudinal study to strengthen our collective understanding of children's exposure pathways to these pathogens (59).

## Data Availability Statement

Study data are available at https://dataverse.harvard.edu/dataset.xhtml?persistentId=doi:10.7910/DVN/CPCK91.

## Ethics Statement

All the study procedures were performed in accordance with the Declaration of Helsinki and ethical approvals were obtained from the Haramaya University Institutional Health Ethics Research Review Committee (Ref. No. IHRERC/152/2018), the Ethiopia National Research Ethics Review Committee (Ref. No. MoST/3-10/168/2018), the Institutional Review Board at the University of Florida (UF) (Ref. No. 201703252), and Washington University School of Medicine (Protocol No. 201806021). The operational district officers and local village chiefs were informed about the objectives of the study, and their consent was obtained through a Community Advisory Board established for the study (60). Written consents were also obtained from both the child's mother/caregiver and father. Material and Data Transfer Agreements (MDTA) were signed between HU and all US-based partners. Export permits to ship biological specimens from Ethiopia to the U.S.A were approved by the Ministry of Science and Higher Education of Ethiopia (Ref. No. SHE/SSM/19.1/008/11/19).

## Author Contributions

AH, SM, JY, WG, MM, KB, YY, and GR conceived and designed the study. SM, NS, AM, and KR designed and implemented the survey. AM, MK, NAm, NAs, and YT supervised sample collection, data collection, and laboratory analysis. YT, LD, and MG the samples. DC and ND cleaned and processed the data. DC analyzed the data, with supervision from AH, SM, and YY. DC, SM, and AH wrote the manuscript. All authors contributed to the revision of the manuscript.

## Conflict of Interest

The authors declare that the research was conducted in the absence of any commercial or financial relationships that could be construed as a potential conflict of interest.
