# Supplementary material for: Campylobacter Colonization, Environmental Enteric Dysfunction, Stunting, and Associated Risk Factors Among Young Children in Rural Ethiopia: A Cross-Sectional Study From the Campylobacter Genomics and Environmental Enteric Dysfunction (CAGED) Project
Source: Front Public Health. 2021 Jan 21;8:615793. doi: 10.3389/fpubh.2020.615793 (PMC7862945; doi:10.3389/fpubh.2020.615793)
Supplement: Supplementary file 1 [file Data_Sheet_1.DOCX]

*S1 Appendix*

**Outlier removal for length measurements**

The average length measurement for each observation was calculated in the following way. We calculated standardized residuals by first calculating the medians (n = 306) of the three measurements for each set of three length observations of the studied children (n = 102). Then, we divided the difference between each length measurement and the median of the three measurements by the standard deviation of the three measurements. We defined outliers as any values that fell outside the (-3, 3) interval of a standard normal distribution (Figure 1). We then removed the outlying lengths and calculated the average length by taking the mean of two or three measurements for each child.


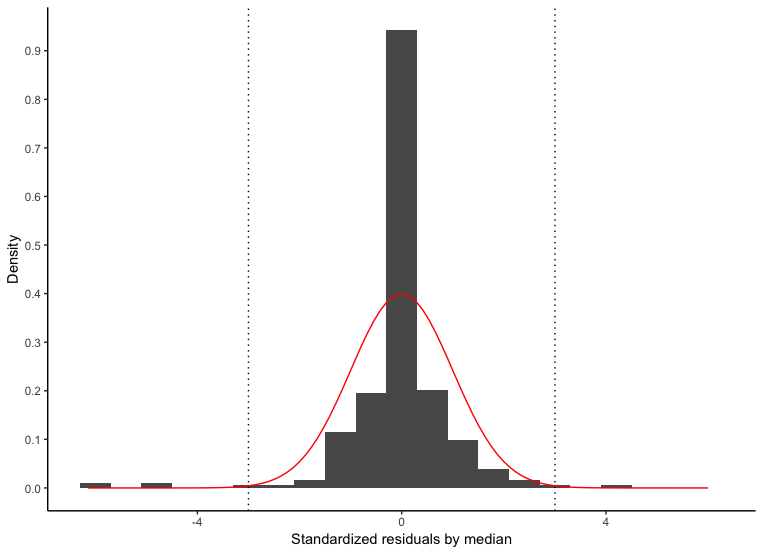


**Figure 1. Distribution of median-centralized and standardized residuals for recumbent length (n=3×102=306).** The histogram shows the density distribution of centralized and standardized length residuals and the standard normal distribution in red (μ = 0, σ = 1).
