# Supplementary material for: Campylobacter Colonization, Environmental Enteric Dysfunction, Stunting, and Associated Risk Factors Among Young Children in Rural Ethiopia: A Cross-Sectional Study From the Campylobacter Genomics and Environmental Enteric Dysfunction (CAGED) Project
Source: Front Public Health. 2021 Jan 21;8:615793. doi: 10.3389/fpubh.2020.615793 (PMC7862945; doi:10.3389/fpubh.2020.615793)
Supplement: Supplementary file 2 [file Table_1.DOCX]

Supplementary Tables and Figures

**S1 Table. Dual sugar absorption test results for 101 children.**

|  | L: R* ratio | % L absorbed | %R absorbed |
| --- | --- | --- | --- |
| Minimum | 0.00 | 0.00 | 0.00 |
| 1^st^ quartile | 0.13 | 0.051 | 1.56 |
| Median | 0.26 | 0.16 | 3.53 |
| Mean | 0.68 | 0.27 | 4.03 |
| 3^rd^ quartile | 0.53 | 0.32 | 5.53 |
| Maximum | 28.9 | 1.36 | 24.9 |

*L: lactulose; R: rhamnose.

**S2 Table. Classification of EED based on dual sugar absorption test and concentration of fecal myeloperoxidase (MPO).**

|  | Normal MPO^1^ | Moderate MPO^2^ | Severe MPO^3^ | Row sum |
| --- | --- | --- | --- | --- |
| Normal  %L ≤ 0.2 | No EED – 27 | No EED – 13 | Moderate EED – 15 | 55 |
| Moderately elevated  0.2 < %L ≤ 0.45 | No EED – 10 | Moderate EED – 12 | Severe EED – 7 | 29 |
| Severely elevated  0.45 < %L | Moderate EED – 6 | Severe EED – 7 | Severe EED – 3 | 16 |
| Column sum | 43 | 32 | 25 | 100 |

^1^ Normal: MPO ≤ 2,000 ng/mL.

^2^Moderately elevated MPO: 2,000 < MPO ≤ 11,000 ng/mL.

^3^Severely elevated MPO: 11,000 ng/mL < MPO ng/mL.

**S3 Table. Anthropometric data for 102 children.**

|  | Mean (SD) | IQR | Range |
| --- | --- | --- | --- |
| Age (days) | 428 (31) | 406/452 | 360/498 |
| Length (cm) | 72.4 (4.3) | 70.0 /74.6 | 59.0/90.0 |
| Weight (kg) | 8.51 (1.20) | 7.79/9.25 | 5.71/12.76 |
| MUAC^1^ (mm) | 130 (11) | 123/138 | 103/157 |
| LAZ^2^ | -1.88 (1.56) | -2.58 /-1.18 | -6.82/4.49 |
| WAZ^3^ | -1.25 (1.18) | -2.08/-0.44 | -4.48/2.02 |
| WLZ^4^ | -0.42 (0.91) | -1.01/0.28 | -3.27/1.75 |

^1^ Middle Upper Arm Circumference.

^2^ Length-for-Age Z score.

^3^ Weight-for-Age Z score.

^4^ Weight-for-Length Z score.

**S4 Table. Associations between *Campylobacter* PCR results and explanatory variables by logistic regression.**

|  | PCR  Positive | PCR  Negative | Crude OR  (p-value) | Adjusted OR^^^  (p-value) |
| --- | --- | --- | --- | --- |
| Child characteristics |  |  |  |  |
| Age |  |  | 0.70 (0.37) | 0.44 (0.09) |
| ≥ 431 days (median) | 24 (0.46) | 28 (0.54) |  |  |
| < 431 days | 27 (0.55) | 22 (0.45) |  |  |
| Sex |  |  | 0.59 (0.19) | 0.55 (0.21) |
| Female | 23 (0.44) | 29 (0.56) |  |  |
| Male | 28 (0.57) | 21 (0.43) |  |  |
| Kebele group |  |  | (< 0.001) | (< 0.001) |
| One (ref.) | 26 (0.84) | 5 (0.16) |  |  |
| Two | 21 (0.42) | 29 (0.58) | 0.14 | 0.11 |
| Three | 4 (0.2) | 16 (0.8) | 0.05 | 0.04 |
| Breastfed at birth |  |  | NA | NA |
| Yes | 50 (0.51) | 49 (0.49) |  |  |
| No | 0 (0) | 1 (1) |  |  |
| Currently breastfed |  |  | 2.98 (0.10) | 9.5 (0.02) |
| Yes | 47 (0.53) | 42 (0.47) |  |  |
| No | 3 (0.27) | 8 (0.73) |  |  |
| Exclusive breastfeeding through 6 months |  |  | 0.88 (0.76) | 0.89 (0.82) |
| Yes | 24 (0.48) | 26 (0.52) |  |  |
| No | 23 (0.51) | 22 (0.49) |  |  |
| Having diarrhea in past 15 days |  |  | 0.70 (0.37) | 0.58 (0.26) |
| Yes | 22 (0.46) | 26 (0.54) |  |  |
| No | 29 (0.55) | 24 (0.45) |  |  |
| Current diarrhea |  |  | 1.43 (0.56) | 2.06 (0.31) |
| Yes | 7 (0.58) | 5 (0.42) |  |  |
| No | 44 (0.49) | 45 (0.51) |  |  |
| Having fever in past 15 days |  |  | 1.17 (0.69) | 0.99 (0.98) |
| Yes | 27 (0.53) | 24 (0.47) |  |  |
| No | 24 (0.49) | 25 (0.51) |  |  |
| Current fever |  |  | 0.63 (0.61) | 0.44 (0.49) |
| Yes | 2 (0.4) | 3 (0.6) |  |  |
| No | 49 (0.52) | 46 (0.48) |  |  |
| Minimum Dietary Diversity |  |  | 0.84 (0.80) | 1.36 (0.69) |
| Adequate (≥ 5) | 4 (0.44) | 5 (0.56) |  |  |
| Insufficient (< 5) | 42 (0.49) | 44 (0.51) |  |  |
| Animal source food consumption in last 24 hours |  |  | 1.43 (0.37) | 4.01 (0.01) |
| Yes | 31 (0.54) | 26 (0.46) |  |  |
| No | 20 (0.45) | 24 (0.55) |  |  |
| Egg consumption in last 24 hrs. |  |  | 4.17 (0.16) | 5.87 (0.10) |
| Yes | 4 (0.8) | 1 (0.2) |  |  |
| No | 47 (0.49) | 49 (0.51) |  |  |
| Maternal characteristics |  |  |  |  |
| Mother’s age |  |  | 1.00 (1.00) | 1.08 (0.87) |
| ≥ 26 year (median) | 26 (0.51) | 25 (0.49) |  |  |
| < 26 year | 25 (0.51) | 24 (0.49) |  |  |
| Animal source food consumption in last 24 hours |  |  | 1.09 (0.84) | 1.45 (0.45) |
| Yes | 19 (0.51) | 18 (0.49) |  |  |
| No | 30 (0.49) | 31 (0.51) |  |  |
| Handwashing ladder (female) |  |  | 0.73 (0.70) | 1.13 (0.88) |
| Limited | 3 (0.43) | 4 (0.57) |  |  |
| No Facility | 47 (0.51) | 46 (0.49) |  |  |
| Household characteristics |  |  |  |  |
| Drinking water |  |  | 0.85 (0.68) | 0.91 (0.86) |
| Basic, or safely managed | 27 (0.48) | 29 (0.52) |  |  |
| Limited | 22 (0.52) | 20 (0.48) |  |  |
| Sanitation |  |  | 1.09 (0.86) | 2.18 (0.20) |
| Limited | 12 (0.52) | 11 (0.48) |  |  |
| Open Defecation | 39 (0.5) | 39 (0.5) |  |  |
| Tropical Livestock Units |  |  | 0.69 (0.36) | 1.04 (0.94) |
| ≥ 1.2 (median) | 26 (0.46) | 30 (0.54) |  |  |
| < 1.2 | 25 (0.56) | 20 (0.44) |  |  |
| Chicken nighttime location |  |  | 1.27 (0.56) | 1.14 (0.78) |
| Kept in house at night (with or without confinement) | 29 (0.54) | 25 (0.46) |  |  |
| No chickens, or not kept inside at night | 22 (0.48) | 24 (0.52) |  |  |
| Wealth (Asset) |  |  | 0.55 (0.14) | 0.96 (0.94) |
| ≥ median | 22 (0.43) | 29 (0.57) |  |  |
| < median | 29 (0.58) | 21 (0.42) |  |  |
| Wealth (Income) |  |  | 0.96 (0.92) | 0.97 (0.95) |
| ≥ median | 25 (0.5) | 25 (0.5) |  |  |
| < median | 26 (0.51) | 25 (0.49) |  |  |

^^^Adjusted for child age, sex and kebele group

**S5 Table. Associations between EED results and explanatory variables by logistic regression.**

|  | EED  Positive | EED  Negative | Crude OR  (p-value) | Adjusted OR^^^  (p-value) |
| --- | --- | --- | --- | --- |
| Child characteristics |  |  |  |  |
| Age |  |  | 2.07 (0.07) | 1.81 (0.16) |
| ≥ 431 days (median) | 30 (0.59) | 21 (0.41) |  |  |
| < 431 days | 20 (0.41) | 29 (0.59) |  |  |
| Sex |  |  | 0.85 (0.69) | 0.91 (0.82) |
| Female | 25 (0.48) | 27 (0.52) |  |  |
| Male | 25 (0.52) | 23 (0.48) |  |  |
| Kebele group |  |  | (0.11) | (0.23) |
| One (ref.) | 19 (0.63) | 11 (0.37) |  |  |
| Two | 20 (0.4) | 30 (0.6) | 0.39 | 0.44 |
| Three | 11 (0.55) | 9 (0.45) | 0.71 | 0.72 |
| *Campylobacter* in child, by PCR |  |  | 1.17 (0.69) | 1.00 (0.99) |
| Positive | 26 (0.52) | 24 (0.48) |  |  |
| Negative | 24 (0.48) | 26 (0.52) |  |  |
| *Campylobacter* by MeTRS |  |  | 2.17 (0.06) | 2.29 (0.06) |
| log_10_(RPM) ≥ median | 30 (0.6) | 20 (0.4) |  |  |
| log_10_(RPM) < median | 20 (0.41) | 29 (0.59) |  |  |
| *C. jejuni* by MeTRS |  |  | 1.22 (0.61) | 1.39 (0.44) |
| log_10_(RPM) ≥ median | 26 (0.53) | 23 (0.47) |  |  |
| log_10_(RPM) < median | 24 (0.48) | 26 (0.52) |  |  |
| Breastfed at birth |  |  | 0 (0.23) | 0 (0.37) |
| Yes | 48 (0.49) | 50 (0.51) |  |  |
| No | 1 (1) | 0 (0) |  |  |
| Currently breastfed |  |  | 1.83 (0.35) | 2.61 (0.16) |
| Yes | 45 (0.51) | 43 (0.49) |  |  |
| No | 4 (0.36) | 7 (0.64) |  |  |
| Exclusive breastfeeding through 6 months |  |  | 1.84 (0.14) | 2.46 (0.05) |
| Yes | 28 (0.56) | 22 (0.44) |  |  |
| No | 18 (0.41) | 26 (0.59) |  |  |
| Having diarrhea in past 15 days |  |  | 1.62 (0.23) | 1.4 (0.43) |
| Yes | 27 (0.56) | 21 (0.44) |  |  |
| No | 23 (0.44) | 29 (0.56) |  |  |
| Having fever in past 15 days |  |  | 1.85 (0.13) | 1.49 (0.36) |
| Yes | 29 (0.57) | 22 (0.43) |  |  |
| No | 20 (0.42) | 28 (0.58) |  |  |
| Current fever |  |  | NA | NA |
| Yes | 5 (1) | 0 (0) |  |  |
| No | 44 (0.47) | 50 (0.53) |  |  |
| Minimum Dietary Diversity |  |  | 0.82 (0.78) | 1.28 (0.75) |
| Adequate (≥ 5) | 4 (0.44) | 5 (0.56) |  |  |
| Insufficient (< 5) | 42 (0.49) | 43 (0.51) |  |  |
| Animal source food consumption in last 24 hours |  |  | 1.09 (0.84) | 1.45 (0.40) |
| Yes | 29 (0.51) | 28 (0.49) |  |  |
| No | 21 (0.49) | 22 (0.51) |  |  |
| Egg consumption in last 24 hrs. |  |  | 1.53 (0.65) | 2.49 (0.35) |
| Yes | 3 (0.6) | 2 (0.4) |  |  |
| No | 47 (0.49) | 48 (0.51) |  |  |
| Maternal characteristics |  |  |  |  |
| Mother’s age |  |  | 1.84 (0.13) | 2.2 (0.06) |
| ≥ 26 year (median) | 29 (0.58) | 21 (0.42) |  |  |
| < 26 year | 21 (0.43) | 28 (0.57) |  |  |
| Animal source food consumption in last 24 hours |  |  | 0.45 (0.06) | 0.51 (0.12) |
| Yes | 14 (0.38) | 23 (0.62) |  |  |
| No | 35 (0.57) | 26 (0.43) |  |  |
| Handwashing ladder (female) |  |  | 0.72 (0.67) | 1.01 (0.99) |
| Limited | 3 (0.43) | 4 (0.57) |  |  |
| No Facility | 47 (0.51) | 45 (0.49) |  |  |
| Household characteristics |  |  |  |  |
| Drinking water |  |  | 0.33 (0.009) | 0.34 (0.02) |
| Basic, or safely managed | 22 (0.4) | 33 (0.6) |  |  |
| Limited | 28 (0.67) | 14 (0.33) |  |  |
| Sanitation |  |  | 1.6 (0.33) | 1.4 (0.54) |
| Limited | 13 (0.59) | 9 (0.41) |  |  |
| Open Defecation | 37 (0.47) | 41 (0.53) |  |  |
| Tropical Livestock Units |  |  | 1.63 (0.23) | 1.7 (0.22) |
| ≥ 1.2 (median) | 31 (0.55) | 25 (0.45) |  |  |
| < 1.2 | 19 (0.43) | 25 (0.57) |  |  |
| Chicken nighttime location |  |  | 1.84 (0.13) | 1.95 (0.11) |
| Kept in house at night (with or without confinement) | 31 (0.57) | 23 (0.43) |  |  |
| No chickens, or not kept inside at night | 19 (0.42) | 26 (0.58) |  |  |
| Wealth (Asset) |  |  | 1.38 (0.42) | 1.48 (0.37) |
| ≥ median | 27 (0.54) | 23 (0.46) |  |  |
| < median | 23 (0.46) | 27 (0.54) |  |  |
| Wealth (Income) |  |  | 0.92 (0.84) | 1.07 (0.87) |
| ≥ median | 24 (0.49) | 25 (0.51) |  |  |
| < median | 26 (0.51) | 25 (0.49) |  |  |

^^^Adjusted for child age, sex and kebele group

**S6 Table. Associations between stunting results and explanatory variables by logistic regression.**

|  | Stunted | Not stunted | Crude OR  (p-value) | Adjusted OR^^^  (p-value) |
| --- | --- | --- | --- | --- |
| Child characteristics |  |  |  |  |
| Age |  |  | 1.29 (0.52) | 1.17 (0.71) |
| ≥ 431 days (median) | 23 (0.44) | 29 (0.56) |  |  |
| < 431 days | 19 (0.38) | 31 (0.62) |  |  |
| Sex |  |  | 1.52 (0.30) | 1.6 (0.26) |
| Female | 24 (0.46) | 28 (0.54) |  |  |
| Male | 18 (0.36) | 32 (0.64) |  |  |
| Kebele group |  |  | 0.37 | 0.37 |
| One (ref.) | 16 (0.52) | 15 (0.48) |  |  |
| Two | 19 (0.37) | 32 (0.63) | 0.56 | 0.54 |
| Three | 7 (0.35) | 13 (0.65) | 0.50 | 0.50 |
| EED |  |  | 0.86 (0.70) | 0.82 (0.64) |
| Yes (%L ≥ 0.2) | 18 (0.39) | 28 (0.61) |  |  |
| No (%L < 0.2) | 24 (0.43) | 32 (0.57) |  |  |
| *Campylobacter* in child, by PCR |  |  | 1.34 (0.47) | 1.14 (0.78) |
| Positive | 23 (0.45) | 28 (0.55) |  |  |
| Negative | 19 (0.38) | 31 (0.62) |  |  |
| *Campylobacter* by MeTRS |  |  | 0.66 (0.31) | 0.58 (0.20) |
| log_10_(RPM) ≥ median | 18 (0.36) | 32 (0.64) |  |  |
| log_10_(RPM) < median | 23 (0.46) | 27 (0.54) |  |  |
| *C. jejuni* by MeTRS |  |  | 1.28 (0.54) | 1.27 (0.57) |
| log_10_(RPM) ≥ median | 22 (0.44) | 28 (0.56) |  |  |
| log_10_(RPM) < median | 19 (0.38) | 31 (0.62) |  |  |
| Breastfed at birth |  |  | NA | NA |
| Yes | 42 (0.42) | 58 (0.58) |  |  |
| No | 0 (0) | 1 (1) |  |  |
| Currently breastfed |  |  | 0.84 (0.78) | 0.94 (0.93) |
| Yes | 37 (0.41) | 53 (0.59) |  |  |
| No | 5 (0.45) | 6 (0.55) |  |  |
| Exclusive breastfeeding through 6 months |  |  | 1.13 (0.78) | 0.94 (0.89) |
| Yes | 21 (0.42) | 29 (0.58) |  |  |
| No | 18 (0.39) | 28 (0.61) |  |  |
| Having diarrhea in past 15 days |  |  | 1.22 (0.62) | 1.13 (0.78) |
| Yes | 21 (0.44) | 27 (0.56) |  |  |
| No | 21 (0.39) | 33 (0.61) |  |  |
| Current diarrhea |  |  | 0.68 (0.55) | 0.78 (0.70) |
| Yes | 4 (0.33) | 8 (0.67) |  |  |
| No | 38 (0.42) | 52 (0.58) |  |  |
| Having fever in past 15 days |  |  | 1.58 (0.26) | 1.41 (0.42) |
| Yes | 24 (0.47) | 27 (0.53) |  |  |
| No | 18 (0.36) | 32 (0.64) |  |  |
| Current fever |  |  | 2.19 (0.40) | 2.26 (0.39) |
| Yes | 3 (0.6) | 2 (0.4) |  |  |
| No | 39 (0.41) | 57 (0.59) |  |  |
| Minimum Dietary Diversity |  |  | 0.68 (0.59) | 0.78 (0.74) |
| Adequate (≥ 5) | 3 (0.33) | 6 (0.67) |  |  |
| Insufficient (< 5) | 37 (0.43) | 50 (0.57) |  |  |
| Animal source food consumption in last 24 hours |  |  | 0.93 (0.85) | 1.07 (0.88) |
| Yes | 23 (0.4) | 34 (0.6) |  |  |
| No | 19 (0.42) | 26 (0.58) |  |  |
| Egg consumption in last 24 hrs. |  |  | 0.34 (0.30) | 0.46 (0.48) |
| Yes | 1 (0.2) | 4 (0.8) |  |  |
| No | 41 (0.42) | 56 (0.58) |  |  |
| Maternal characteristics |  |  |  |  |
| Mother’s age |  |  | 0.82 (0.63) | 0.88 (0.76) |
| ≥ 26 year (median) | 20 (0.39) | 31 (0.61) |  |  |
| < 26 year | 22 (0.44) | 28 (0.56) |  |  |
| Animal source food consumption in last 24 hours |  |  | 1.21 (0.66) | 1.29 (0.56) |
| Yes | 16 (0.43) | 21 (0.57) |  |  |
| No | 24 (0.39) | 38 (0.61) |  |  |
| Handwashing ladder (female) |  |  | 0.56 (0.49) | 0.7 (0.68) |
| Limited | 2 (0.29) | 5 (0.71) |  |  |
| No Facility | 39 (0.41) | 55 (0.59) |  |  |
| Household characteristics |  |  |  |  |
| Drinking water |  |  | 0.64 (0.28) | 0.68 (0.38) |
| Basic, or safely managed | 21 (0.37) | 36 (0.63) |  |  |
| Limited | 20 (0.48) | 22 (0.52) |  |  |
| Sanitation |  |  | 1.13 (0.80) | 1.14 (0.81) |
| Limited | 10 (0.43) | 13 (0.57) |  |  |
| Open Defecation | 32 (0.41) | 47 (0.59) |  |  |
| Tropical Livestock Units |  |  | 0.71 (0.41) | 0.69 (0.39) |
| ≥ 1.2 (median) | 21 (0.38) | 35 (0.62) |  |  |
| < 1.2 | 21 (0.46) | 25 (0.54) |  |  |
| Chicken nighttime location |  |  | 0.67 (0.32) | 0.64 (0.28) |
| Kept in house at night (with or without confinement) | 20 (0.37) | 34 (0.63) |  |  |
| No chickens, or not kept inside at night | 22 (0.47) | 25 (0.53) |  |  |
| Wealth (Asset) |  |  | 0.61 (0.23) | 0.61 (0.25) |
| ≥ median | 18 (0.35) | 33 (0.65) |  |  |
| < median | 24 (0.47) | 27 (0.53) |  |  |
| Wealth (Income) |  |  | 1.00 (1.00) | 0.98 (0.96) |
| ≥ median | 21 (0.41) | 30 (0.59) |  |  |
| < median | 21 (0.41) | 30 (0.59) |  |  |

^^^Adjusted for child age, sex and kebele group


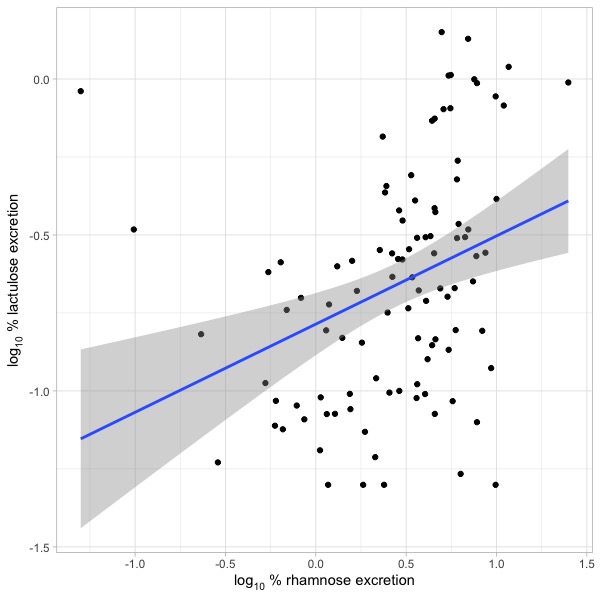


**S1 Figure. Correlation between excretion of lactulose and rhamnose.**
